# Negative health care experiences of immigrant patients: a qualitative study

**DOI:** 10.1186/1472-6963-11-10

**Published:** 2011-01-14

**Authors:** Jeanine Suurmond, Ellen Uiters, Martine C de Bruijne, Karien Stronks, Marie-Louise Essink-Bot

**Affiliations:** 1 Academic Medical Centre/University of Amsterdam, Department of Social Medicine. Amsterdam, The Netherlands; 2 The National Institute for Public Health and the Environment (RIVM), Bilthoven, The Netherlands; 3 EMGO Institute - Vumc, Department of public & occupational health, Amsterdam, The Netherlands

## Abstract

**Background:**

Negative events are abusive, potentially dangerous or life-threatening health care events, as perceived by the patient. Patients' perceptions of negative events are regarded as a potentially important source of information about the quality of health care. We explored negative events in hospital care as perceived by immigrant patients.

**Methods:**

Semi-structured individual and group interviews were conducted with respondents about negative experiences of health care. Interviews were transcribed and analyzed using a framework method. A total of 22 respondents representing 7 non-Dutch ethnic origins were interviewed; each respondent reported a negative event in hospital care or treatment.

**Results:**

Respondents reported negative events in relation to: 1) inadequate information exchange with care providers; 2) different expectations between respondents and care providers about medical procedures; 3) experienced prejudicial behavior on the part of care providers.

**Conclusions:**

We identified three key situations in which negative events were experienced by immigrant patients. Exploring negative events from the immigrant patient perspective offers important information to help improve health care. Our results indicate that care providers need to be trained in adequately exchanging information with the immigrant patient and finding out specific patient needs and perspectives on illness and treatment.

## Background

Patients are considered a potentially important source of information about problems or incidents within health care [1]. Their observations can be used in health care quality and safety improvement initiatives [1]. While it has been recognized that not all patients may be able or willing to identify problems and incidents in their care, errors detected by patients are not easily identified by other means such as reports from health professionals or medical record reviews [2]. Patients' perceptions of problems and incidents may thus add to a broader understanding of problems in in-patient care [3]. Patients may be particularly capable to report on interpersonal problems, such as lack of respect or insufficient information, and on incidents related to the delivery of care. Patients may, however, be less likely to notice deficiencies in technical quality of health care and in the appropriateness of medical decisions [4]. Research on the patient perspective on problems and incidents in health care rarely includes ethnic minority patients. There is, as Johnstone and Kanitsaki [5] put it, a "paucity" of research on the experiences with problems and incidents of patients from diverse cultural and language backgrounds. That it may be important to include ethnic minority patients' perceptions of problems and incidents within health care, is indicated by the large amount of studies that document racial/ethnic disparities in the diagnosis and treatment of various conditions [6]. Language differences between care provider and patient as well as the use of family or friends as interpreters instead of professional interpreters can lead to errors and incidents, with potentially serious clinical consequences [7-10]. Bias, stereotyping, prejudice, and clinical uncertainty on the part of health care providers may contribute to racial and ethnic disparities in health care. Important safety incidents in the eyes of ethnic minority patients were those in which racism or prejudice occurred [11]. Garrett et al. found in their study that immigrant patients in the Emergency Department experienced negative events in relation to staff neglect, communication and language issues, as well as medication and diagnostic errors [12].

In order to get more insight in the situations in which patients have negative experiences with health care, we explored in this paper the perceptions of immigrant patients in hospital care and treatment. In contrast to other researchers' findings that patients reports are a reliable method to identify error and harm in medical practice [1], we believed that patient's perceptions of an event may not necessarily match the views of the care provider, and therefore cannot be termed as an adverse event, a patient safety incident or an error. We will use in this paper the term 'negative event' to refer to an abusive, potentially dangerous or life-threatening health care event, as perceived by the patient [12]. We want to identify the specific situations in which immigrant patients perceived negative events in hospital care and treatment.

## Methods

### Design

This qualitative study was conducted using semi-structured interviews involving immigrant respondents. In the interview, they described a negative event from their own or friend/family member's experience. We used the critical incident technique, in which participants are asked to describe an event about a remarkable incident [13,14]. This technique helps to obtain rich contextual information, as it uncovers tacit knowledge by allowing participants to describe what their thought processes and actions were during the specific event. In the present study, respondents were asked to recall a remarkable incident in which, in their perception, something occurred during their medical care or treatment that they had not expected, or that they found unusual or inappropriate.

### Respondents

Respondents included a convenience sample of 22 immigrants living in the Netherlands. We first contacted by email our own network of health care providers previously established by our group for other research projects, and asked whether they knew anyone who had experienced anything unusual or inappropriate in their in-hospital medical care. Care providers either did not know such a respondent or the respondent they approached did not want to cooperate because, for example, the experience had been too painful. Our request finally resulted in one person: namely, the daughter of a male respondent who had experienced severe problems within the hospital, but who did not want to be interviewed personally because the memory was too painful for him to talk about. We then approached by email about 20 contact persons in different local immigrant organizations. They were asked whether they knew anyone who had experienced anything unusual or inappropriate in their in-hospital medical care. Local immigrant organizations in the Netherlands are usually small, and they help their members with specific needs or problems. The chances were therefore high that the contact persons would know someone who had experiences with in-hospital care.

We received responses from a Chinese, a Portuguese, and two Spanish-speaking immigrant organizations. With their consent, we were able to approach two Chinese respondents, one Portuguese respondent, a respondent from Chili, and a respondent from the Dominican Republic, respectively, for an interview. In addition, we talked to a local organization that takes care of the elderly from different ethnic backgrounds. This resulted in two contact persons: An Italian contact person who approached one person, and also wanted to be interviewed himself; and a Turkish contact person who knew of a small Turkish group of women meeting every week to discuss all kinds of issues involving health and health care. The mediator of this group was approached and consented to a group interview. Before the group interview took place, all members present agreed to it.

As is common in the Netherlands, the respondent's ethnic origin was classified in terms of the person not having been born in the Netherlands, or having at least one parent not born in the Netherlands [15]. The interviewed respondents were of Turkish, Chinese, Italian, Dominican, Chilean, Portuguese, and Surinamese origin (additional file 1 summarizes the respondents' characteristics). The majority of respondents had limited knowledge of the Dutch language, and most were middle-aged first-generation immigrants. Initially, there were 20 respondents, but two interpreters we used for the interviews (see next paragraph on data collection) also recalled a critical incident; the Turkish interpreter seemed to have felt safe in the group interviews to join the group discussion with her own experiences, and the Chinese interpreter recalled a critical incident involving somebody else who did not want to be interviewed because of the painful memories she still had about the event. Because of the richness of information regarding both critical incidents reported by the interpreters, it was decided to include these as well, which adds up to a total sample of 22 respondents who were interviewed.

### Ethical considerations

According to Dutch law, medical ethical approval for this study was not required. Nevertheless, a considerable effort was made to adequately deal with ethical considerations: for example, the anonymity of the respondents and their data was guaranteed by the use of codes, and *a priori *informed consent was obtained from all study participants.

### Data collection

Data were collected over a 4-month consecutive period in 2008-2009. We conducted 11 interviews in total (additional file 1 summarizes the respondents' characteristics in combination with the interview method). Of the 22 respondents, nine were interviewed individually, and two group interviews were held with seven and six (all Turkish women) respondents, respectively. For the Chinese and Turkish interviews we used interpreters. The (both female) interpreters we used were the contact persons and knew the interviewees; we also felt they could create a safe atmosphere for the interview. As mentioned above, two interpreters also joined the interview with their experiences. Seven interviews were conducted in Dutch with individual participants, and 15 participants were interviewed via a Turkish (in the group interviews) or Chinese interpreter (in two individual interviews). The two interpreters told about experiences in Dutch. Interviews were conducted in a location preferred by the interviewees: for instance, at home or in a community centre. Three individual interviews were conducted by telephone at the request of the respondents. Another respondent requested that his daughter be interviewed because he found it too stressful to repeat what had occurred. We used different kinds of individual interviews, such as face-to-face or telephone, and one interview with a daughter of a respondent. Part of the data was collected by means of a 'natural group discussion', defined as a group interview with people who know each other already [16]. We decided to use this combination of methods for four reasons. First, the group setting may generate a safer environment for discussing painful or precarious experiences than a face-to-face interview with an unknown interviewer. Similarly, an interview by telephone at the request of the respondent as well as an interview with a daughter instead of the patient himself may have generated a safer environment to discuss difficult experiences. Second, the use of group interviews enabled us to consult with a wider range of individuals within the resource constraints of the project. Third, interviews with natural groups provided a pragmatic solution in regard to data collection, since many individuals were already part of pre-existing groups and were more likely to participate by way of these. Finally, we achieved triangulation of the method of data collection. This involved collecting data from different levels of persons, individuals, groups, and collectives with the aim of validating data through multiple perspectives on the phenomenon, in order to enhance credibility of the results [17].

All respondents were interviewed using a semi-structured list of six questions (see box 1). Interviews lasted from about 20 minutes (most of the telephone interviews) up to 2 hours (the group interviews), and were conducted by the first author (JS). Of the 9 individual interviews, 6 were face-to-face and tape-recorded, and 3 were telephone interviews during which notes were made. All interviews were subsequently transcribed verbatim by the first author (JS) and an assistant. Only the Dutch fragments were transcribed.

### Data analysis

We used the framework method to analyze the transcribed interviews [18,19]. Framework analysis developed for applied qualitative research, takes a deductive approach informed by a priori reasoning and is more structured than other qualitative methods. This approach involves a systematic process of sifting, charting, and sorting material according to key issues and themes, and has five stages: familiarization with the data, identification of a thematic framework, indexing, charting, and mapping/interpreting. The first author executed all stages of the analysis. First the material was read and reviewed carefully. During this familiarization stage, an overview of the richness and diversity of the data was gained, and was started with the process of abstraction and conceptualization. While the data were reviewed, short notes were made about the range of responses to our questions, as well as about recurrent themes and issues that seemed to be important to respondents, for example language problems played a crucial for many of them. Once the material had been reviewed, the researcher identified in these notes key issues and themes according to which the data could be examined. This resulted in an initial thematic framework of several topics, such as felt language problems, feelings of care providers who behaved 'strange' in the eyes of respondents and feelings of prejudice. We constructed three major subject charts: 1) perceptions of the exchange of information between care provider and patient; 2) perceptions of care providers' perspectives on the patient's illness and treatment; and 3) experienced discrimination and prejudice on the part of care providers. In the next stage, the framework was systematically applied to the material, and all data were reread and annotated accordingly. Text passages were coded, grouped by code and then all passages with a particular code were re-read to identify subthemes. The coding was done manually by the first author, using a code book. Finally, on the basis of these charts, patterns and connections could be described through an iterative and comparative process of searching, reviewing, and comparing the data. To identify possible discrepancies, the first and last author repetitively discussed and continuously refined the analysis.

## Results

Three types of situations were identified in which respondents experienced negative events: 1) situations in which respondents felt that the exchange of information between themselves and the care provider was inadequate; 2) situations in which different expectations between respondents and care providers about medical procedures played a role; and 3) situations in which respondents felt mistreated because of the care provider' prejudicial behavior. These types of situations were not mutually exclusive, and some overlap occurred between them. The analysis did not imply any absolute differences between the types of situations, but merely described several possible conditions within the medical care and treatment. All negative events were categorized into one of the three types of situations; we choose for the most dominant pattern that followed from the analysis. This means that a respondent's description of a negative event was categorized into one dominant type of situation, because that was the dominant pattern, but some respondents also briefly described something that happened that could be categorized into one of the other types of situations.

### Situation 1: Respondents felt that exchange of information was inadequate

Several respondents claimed they had not received sufficient information from the care provider about their medical condition. For example, a Dominican respondent who was in the gynecology ward for laparoscopic surgery on her uterus reported that she was unaware after the operation that an indwelling urethral stent had been put in place; this only became evident after the stent became infected and caused her severe pain (see additional file 2, respondent 4). In addition, her bladder was accidentally perforated during laparoscopic surgery:

*I don't speak Dutch well, and if you're in pain then it's easier to speak Spanish. The doctors thought something had happened, and they didn't tell me. There was a complication, and I wasn't told about it. And I had an indwelling stent I wasn't informed about. I'm still in pain now, and I have totally lost trust*.

This respondent related the negative event explicitly to not being informed well. She explained this by referring to her bad Dutch proficiency. Several other respondents also experienced situations in which they were not informed by care providers about their medical condition. A Chinese respondent told that she was very surprised that after a hysterectomy only her uterus was removed and not the ovaries and the cervix, as she thought her doctor had explained to her.

Other respondents felt the information given by themselves to the care provider was not listened to or not understood. For example, a Chinese respondent scheduled for gynecological surgery to remove a leiomyoma asked her daughter to translate and inform the care providers that she could not undergo anesthesia without an examination because of an existing heart condition. On her way to the operating theatre she repeated this again to a nurse in her very limited Dutch, after which the operation was cancelled (see additional file 2, respondent 2). The patient elaborated on this, see the following excerpt:

Interviewer: So actually it went wrong because of lack of communication?

*Patient, through interpreter: Yes. On the one hand, the daughter of the woman communicated the information, but on the other hand, the care providers did not read the patient record. They did not pay attention. Hence, the woman does not think her care was taken seriously*.

Interviewer: How could this be improved?

*Patient through interpreter: Before an operation, care providers should read the patient record carefully and take into account the special conditions of the patients. Also, even though patients' children can come to the hospital to interpret, the woman would have preferred a formal interpreter*.

This respondent felt that bad communication contributed to the negative event. She also felt that care providers had not read her patient record, nor 'take into account special conditions of patients', which led her believe that care providers did not take her care seriously. In contrast to the Dominican respondent (additional file 2, respondent 4), this respondent believed that the incident was not caused by her failure to speak Dutch but by a shortcoming of the hospital. She suggested using formal interpreters instead of family members. In the Turkish group interviews, several respondents mentioned too that they would prefer a formal interpreter to a family member, because family members could inadvertently fail to correctly interpret the "medical" conversation with the care provider. For example, a Turkish respondent (additional file 2, respondent 16) who generally took her son as an interpreter when she went to the doctor stated:

If there are three things I only tell two. And the doctor says: "I understand you well". But how can he understand me if I have the idea that I can't explain myself well?

This respondent reflected that she was withholding information from the doctor when she took her son as an interpreter. However, she felt she was not taken seriously by the doctor because - in her eyes - he did not refer to the inadequate information exchange.

In summary, respondents who experienced a negative event in relation to inadequate exchange of information related this to language differences between themselves and the care provider. Whereas one respondent felt this was her fault, other respondents believed that it was related to the choice of interpreter. Interestingly, all patients felt themselves responsible for the interpretation of the consultation. No one knew that in the Netherlands this is the responsibility of the care provider (interpretation services are free for care providers and they only have to make a phone call to make a reservation). Because of the inadequate information exchange, respondents experienced mistrust in the care provider or felt that their medical care was not taken seriously.

### Situation 2: Respondents experienced a difference in expectations between themselves and care providers about medical procedures

In this type of situation, respondents experienced a difference in expectations between themselves and care providers about medical procedures. Respondents formulated this in terms of a care provider who did unexpected and/or illogical things. Either respondents believed that something was very wrong with their medical condition but the care provider did nothing; or respondents believed that the care provider offered treatment that the respondent thought was unnecessary. This situation was primarily expressed by participants of the Turkish interview groups, although other participants referred briefly to it as well. For example, a Turkish respondent (additional file 2, respondent 13) reported that she went to the emergency room because she felt extremely nauseous and thought she had eaten something poisonous. To her surprise, she had to wait 5 hours before she was seen by a doctor; in the meantime, she was extremely anxious because she felt the poison should be "taken out" as soon as possible.

Some respondents complained about seemingly small issues in the light of a severe medical complication: for example, the Dominican respondent whose bladder was accidentally perforated (additional file 2, respondent 4). The day after the operation, her treating physician went abroad to attend a conference. The respondent could not understand how her doctor could leave her after making a medical error; she felt abandoned.

Other respondents believed that a certain treatment was not necessary but to their surprise it was offered to them. For example, another Turkish respondent related that she was admitted to hospital with what she thought was food poisoning (additional file 2, respondent 12). To her astonishment, she underwent an operation and, at the time of the present study, still does not understand why she needed an operation to treat food poisoning. A Chinese respondent reported that she had had several operations (additional file 2, respondent 1), and that a procedure to remove a myoma from her uterus was scheduled; however, she postponed it because, as the interpreter explained:

*She was told by her brother that a person can only have anesthesia six times during a lifetime. She had already had five operations, and did not want a sixth. [...] She wanted to save the last operation in case of something extremely serious*.

From her perspective, this respondent had only one operation left, and she wanted to keep this for a specific occasion. This is in total contrast to the viewpoint of the surgeon, who saw no problem in performing the operation.

In summary, the respondents reported negative events in relation to care providers who acted in their eyes illogical and unexpected, and therefore felt that care providers did not act in their interests. The difference in perspectives may be explained by language problems between care provider and patient, when the care provider was unable to adequately explain the biomedical perspective to the patient. It also can be referred to as a difference between an 'illness perspective' of the patient and a 'disease perspective' of the care provider [20]. The explanatory models that respondents used to explain their illness, may bear little relation to those of the care providers, and it is unclear to what extent the respondents shared their perspective with the care provider. Only the Chinese respondent (additional file 2, respondent 1) explicitly referred to conflicting perspectives between herself and her doctor.

### Situation 3: Respondents felt excluded from optimal care because care providers were prejudiced or discriminate

Several respondents felt that prejudice on the part of care providers played a role. One example was a Surinamese patient (additional file 2, respondent 21) whose postoperative complaints were not taken seriously; two days after the operation it was discovered he had a perforated oesophagus and had to be transferred to Intensive Care. In the interview, his daughter explained that she thought care providers' prejudice concerning how ethnic minorities express pain may have played a role in their neglect of the respondent's complaints:

*I believe they thought it was a different perception of illness, and that was the reason the complaints were not taken seriously. [...] [Interviewer: And what may the different illness perception have been? ] That patients with an ethnic minority background have an illness perception that is more dramatic, more severe, more profound... This presumption may have played a role*.

This respondent was the only one who was fluent in Dutch, and his daughter was highly educated and able to reflect very clearly on the negative event. This was the only negative event in which it was certain there had not been a language problem between care provider and patient. In this case the daughter also explained that an official complaint was lodged with the complaints committee of the hospital. To their astonishment the hospital refused to admit that mistakes were made.

This respondent related the negative event to prejudicial behaviour of care providers and saw that as a reason why complaints were not taken seriously by care providers. Several other respondents felt that the negative event was related to neglect. They felt that they, in contrast to Dutch patients, had to wait longer in the waiting room or for a certain treatment. For example, the Chinese respondent (additional file 2, respondent 2) whose operation was cancelled was told to wait for an examination by a cardiologist. Despite waiting for several hours, she was given no food or liquids, even though she was hungry and thirsty after having fasted since the previous night. She felt neglected, and believed she did not receive the same quality of care as Dutch patients. She attributed this to the low quality of the communication:

Interviewer: Did you feel discriminated against?

*Patient through interpreter: A little bit*.

Interviewer: Do you think it would happen less with Dutch patients?

*Interpreter: Yes, it would happen less with Dutch patients because of the communication*.

This respondent felt that if she would have spoken Dutch well she could have expressed her needs better, for example she could have asked herself for something to eat or drink. These respondents may have perceived inadequacies in services as based on discrimination, when the problem may have been a generally poor service as well as communication failure to explain these services.

## Discussion

Immigrant patients reported negative events in three types of situations: 1) inadequate exchange of information between care provider and patient; 2) unexpected differences between the care provider's and the patient's perspectives with regard to disease and management; and 3) experienced prejudicial behavior on the part of care providers. Experiencing strange or unexpected events may also lead to low trust (as explicitly expressed by some respondents). Low trust has been shown in the literature to be associated with lower satisfaction with care, lower adherence to treatment recommendations, worse self-reported health, reduced willingness to seek care, and lower-quality relationship with care providers [21-24].

Exploring negative events from the immigrant patient perspective offers important new information to help improve health care. Many of the incidents detected by the patients in our study can be classified as quality problems, as also Weingart et al. reported in their interview study on patients' ability to recognize medical errors [25]. Many of the reported negative events in our study are therefore similar to those reported by non-immigrant patients, and are related to interpersonal problems, such as lack of respect or insufficient information [4]. However, our study also visibly illustrated supplementary problems experienced by immigrant patients i.e. immigrant-specific problems, such as the impact of care providers who did not use professional interpreters. In line with other studies, patients were very clear that they preferred a professional interpreter. Macfarlane et al. also showed a preference of patients for professional interpreters [26]. Hoopman et al. reported that half of the Turkish and Moroccan family interpreters translated information provided by the doctor in a way they thought was less confronting for the patient [27]. A review by Karliner et al. showed that the use of professional interpreters improved the clinical care for patients with limited English proficiency [28].

### Strengths and limitations of the study

Recruitment of immigrant groups can be challenging as they can be 'hard to reach' for study purposes. An important strength of the present study is that even though talking about negative events turned out to be a difficult and sensitive topic, immigrant respondents were willing to share painful, intimate, and personal experiences with us. Respondents obviously felt safe to share their experiences with us, and this has resulted in a rich set of data.

A potential limitation of our study may be that we reported the experiences of the respondents, but do not know what took place from the viewpoint of the care providers. Patients are often less likely to notice deficiencies in technical quality of health care and in the appropriateness of medical decisions [4]. For example, the respondent (additional file 2, respondent 12) who believed she unnecessarily underwent surgery to treat her assumed food poisoning may not have understood (due to communication problems, or limited knowledge of human anatomy) that she had been diagnosed with, for instance, appendicitis. Thus the experiences of patients may not have reflected what in the eyes of doctors happened, and because we did not interview the treating doctor nor had insight in the medical record, we are not sure whether doctors also believed there was a negative event. In future research, we recommend to interview patients and care providers about the same perceived incident or to include the medical record of the patient in the study, in order to be able to compare different perspectives [1]. Another potential limitation may have been in the selection of the respondents. We adopted a convenience sample, including everyone who was willing to participate. Furthermore, we made use of natural groups and had little control over size and composition of the group. This way of recruitment may have resulted in bias, as some members of the group interviews may have experienced the pressure to conform or may have been stifled rather than stimulated by the group.

We, however, believe that this study could not have been executed if we had not chosen a convenience sample and used the natural groups. It is mainly because we were flexible with our methods that we were able to provide a safe environment for respondents to discuss painful experiences, which resulted in extensive data.

### Implications

Specific training for individual care providers might focus on exploring patients' expectations, difficulties, needs and worries, as well as being sensitive to the ways in which patients from other cultures and health care systems may experience a new health care system. Care providers could put more effort into learning patients' beliefs about their illness and its management, because patients may not always spontaneously share this information. They should be aware of the effects when patient beliefs are not well explored, resulting in patients who do not feel taken seriously, who feel low trust in care providers or believe they received low quality care. Furthermore, to increase the adequate exchange of information care providers need to be trained to work with professional interpreters, and instructed in other culturally competent behaviors [28-30]. That this is still important shows a study by Diamond et al. that revealed that medical residents found it easier to "get by" without a professional interpreter even though they were aware of negative implications for the quality of care [31]. In addition, training future doctors need to diminish the effects of racism or unconscious bias related to clinical decision-making [32]. Moreover, patients could be better informed about what they can expect from care and treatment: for example, hospitals should have signage and informative literature in the patient's own language [33]. Providing empowerment training to patients may also help patients to identify and organize questions about diagnosis and treatment, thereby improving their skills and confidence in communicating with care providers [22].

## Conclusions

The present results suggest immigrant patients experience negative events in three types of situations: namely, perceived inadequate exchange of information, experienced difference in expectations between respondents and care providers about medical procedures; and feelings of prejudicial behavior on the part of the care providers. Specific training for individual care providers might enable them to communicate in ways to adequately exchange information and find out specific patient needs as well as perspectives on illness and treatment.

## Competing interests

The authors declare that they have no competing interests.

## Authors' contributions

JS conducted the interviews, analyzed and interpreted the data, and wrote the article. EU, MB, KS, and ME contributed to the analysis, the interpretation of the data, and the preparation of the article. MB and ME originated and designed the study. ME supervised the study, assisted with the article, and was the guarantor. All authors read and approved the final manuscript.

## Pre-publication history

The pre-publication history for this paper can be accessed here:

http://www.biomedcentral.com/1472-6963/11/10/prepub

## Supplementary Material

Additional file 1 **Table S1 Characteristics of respondents negative health care experiences of immigrant patients Suurmond January 2011 **A table with characteristics of respondents (country of birth, age, sex) and the interviews with them (interview method, language of the interview) Click here for file

Additional file 2 **Box 1 Interview guide negative health care experiences of immigrant patients Suurmond January 2011 **A box with the interview guide (6 interview questions) Click here for file

Additional file 3 **Table S2 Description of negative event Negative health care experiences of immigrants Suurmond January 2011 **A table with 22 descriptions of negative events as experienced by immigrant patients Click here for file
