# Supplementary material for: Negative health care experiences of immigrant patients: a qualitative study
Source: BMC Health Serv Res. 2011 Jan 14;11:10. doi: 10.1186/1472-6963-11-10 (PMC3029223; doi:10.1186/1472-6963-11-10)
Supplement: Additional file 1 — Table S1 Characteristics of respondents negative health care experiences of immigrant patients Suurmond January 2011 A table with characteristics of respondents (country of birth, age, sex) and the interviews with them (interview method, language of the interview) [file 1472-6963-11-10-S1.DOC]

Table 1 – Characteristics of respondents and of the interviews with them

| Respondent | Country of birth | Age | Sex | Interview method | Language  of interview |
| --- | --- | --- | --- | --- | --- |
| 1 | Chinese | 53 | Female | Individual face-to-face interview  with help of interpreter | Mandarin |
| 2 | Chinese | 56 | Female | Individual  face-to-face interview  with help of interpreter | Mandarin |
| 3 | Chinese | 58 | Female | Individual face-to-face  interview  with the interpreter | Dutch |
| 4 | Dominican | 52 | Female | Individual face-to-face interview | Dutch |
| 5 | Italian | 60 | Male | Individual face-to-face interview | Dutch |
| 6 | Portuguese | - | Male | Individual interview by  telephone | Dutch |
| 7 | Turkish | Between  45-65 | Female | 1^st^ Group interview with help of interpreter | Turkish |
| 8 | Turkish | Between  45-65 | Female | 1^st^ Group interview with help of interpreter | Turkish |
| 9 | Turkish | Between  45-65 | Female | 1^st^ Group interview with the interpreter | Dutch |
| 10 | Turkish | Between  45-65 | Female | 1^st^ Group interview with help of interpreter | Turkish |
| 11 | Turkish | Between  45-65 | Female | 1^st^ Group interview with help of interpreter | Turkish |
| 12 | Turkish | Between  45-65 | Female | 1^st^ Group interview with help of interpreter | Turkish |
| 13 | Turkish | Between  45-65 | Female | 2^nd^ Group interview with help of interpreter | Turkish |
| 14 | Turkish | Between  45-65 | Female | 2^nd^ Group interview with interpreter | Turkish |
| 15 | Turkish | Between  45-65 | Female | 2^nd^ Group interview with help of interpreter | Turkish |
| 16 | Turkish | Between  45-65 | Female | 2^nd^ Group interview with help of interpreter | Turkish |
| 17 | Italian | - | Male | Individual  Interview  Telephone | Dutch |
| 18 | Chilean | - | Male | Individual interview by  telephone | Dutch |
| 19 | Turkish | Between  45-65 | Female | 1^st^ Group interview with help of interpreter | Turkish |
| 20 | Turkish | Between  45-65 | Female | 2^nd^ Group interview with help of interpreter | Turkish |
| 21 | Surinamese | 61 | Male | Individual face-to-face interview  with daughter | Dutch |
| 22 | Turkish | Between  45-65 | Female | 2^nd^ Group interview with help of interpreter | Turkish |
