# Supplementary material for: Negative health care experiences of immigrant patients: a qualitative study
Source: BMC Health Serv Res. 2011 Jan 14;11:10. doi: 10.1186/1472-6963-11-10 (PMC3029223; doi:10.1186/1472-6963-11-10)
Supplement: Additional file 2 — Box 1 Interview guide negative health care experiences of immigrant patients Suurmond January 2011 A box with the interview guide (6 interview questions) [file 1472-6963-11-10-S2.DOC]

Box 1- Interview guide

1) Can you describe from your experience anything that was unexpected, unusual, or inappropriate in your medical care or treatment?

2) What preceded and contributed to the event?

3) What did you do or not do? How did you feel?

4) What was the outcome or result?

5) In what way did your ethnic or cultural background play a role?

6) How might the described event be avoided in the future?
