# Supplementary material for: Negative health care experiences of immigrant patients: a qualitative study
Source: BMC Health Serv Res. 2011 Jan 14;11:10. doi: 10.1186/1472-6963-11-10 (PMC3029223; doi:10.1186/1472-6963-11-10)
Supplement: Additional file 3 — Table S2 Description of negative event Negative health care experiences of immigrants Suurmond January 2011 A table with 22 descriptions of negative events as experienced by immigrant patients [file 1472-6963-11-10-S3.DOC]

Table 2 - Description of negative event as experienced by respondents

| Respondent | Description of perceived negative event |
| --- | --- |
|  | Situation 1: Respondents felt that exchange of information was inadequate |
| 1 | Chinese respondent said that together with the gynecologist she had decided to have both her uterus and ovaries removed because she suffered from heavy menstrual bleeding. After the operation, she found to her surprise that she had only had a hysterectomy. She was annoyed that she had not been not informed about this beforehand. She was also annoyed that a few years later she had a lot of problems with her ovaries and needed several operations to remove myomas. |
| 2 | Chinese respondent related that she had been scheduled for gynecological surgery to remove a leiomyoma, and she asked her daughter to translate and inform the care providers that she could not undergo anesthesia without an examination because of an existing heart condition. On her way to the operating theatre, she repeated this information again to a nurse in her very limited Dutch, after which the operation was cancelled. |
| 3 | The interpreter related an incident involving a Chinese woman who had been pregnant, and just before the delivery, she needed a caesarean section, “otherwise the baby cannot be saved”. After the operation, the baby died anyway. Twenty years later, the woman suddenly began to have stomach pains. She was examined. In the hospital, and was told that a piece of intestine had been removed during the caesarean section. This had never been told to her. The woman and her husband were very angry because not only had their baby died but no one had ever informed them about the removal of the piece of intestine. |
| 4 | Respondent from the Dominican Republic had been given the choice between laparoscopic surgery on her uterus or a normal operation. She chose the laparoscopic surgery, but the gynecologist “accidentally made a hole in my bladder”. Another operation followed to repair the bladder. The day after the operation, her treating physician went abroad to attend a conference. The respondent could not understand how her doctor could leave her after making a medical error; she felt abandoned. She was also unaware after the operation that an indwelling urethral stent had been put in place; this only became evident after the stent became infected and caused her severe pain. The respondent blamed it on her low proficiency in Dutch. |
| 5 | Italian respondent said he had had a lump (“a fatty thing”) near his throat, which was growing bigger, and on the advice of the GP he decided to have it removed in the hospital. After a few weeks he asked his GP to read a note from the hospital; he then learned that his lymph glands had been removed. Respondent was “a little angry” that no one had told him that. |
|  | Situation 2: Care providers act in unexpected and illogical ways |
| 6 | Portuguese respondent said he had lung cancer and was operated upon. During the operation, he had a cardiac arrest, and subsequently was brought to the intensive care unit. During the night, several nurses and doctors visited him to give him an injection for the pain. When he refused, they told him that they would kill him. The next morning, the respondent told the doctor what had happened. The doctor said that he must have been hallucinating. The respondent thought so too, but he was and still is not sure. |
| 7 | Turkish respondent said that she had had an operation on her gall bladder. After the operation, the hospital called and told to come in for the operation; she then informed them that she had already had the operation. She was told that there had been a mistake in the paper work. She felt this was something very dangerous, because if she had not said she had already had the operation, she would have been operated upon without a reason. |
| 8 | Turkish respondent said she had been pregnant, and was told that she could have “an injection in her stomach to see whether her baby is healthy” (punction). In her passport, the date of birth was such that her age showed 37, but in reality she was only 34. She thus did not need an injection (in the Netherlands every pregnant woman older than 36 can have prenatal screening). She also simply did not want an injection, because she considered it was too dangerous. She delivered a healthy baby and was glad she never had the procedure |
| 9 | Turkish respondent said she had delivered her baby in the hospital but “the placenta did not want to come out”. She had an injection every day to stimulate ejection of the placenta. After a few days a “big red bloody thing” came out, and to her surprise it was the placenta. The respondent was happy that now she could go home, but also felt neglected and thought it was strange the doctors did not do anything to speed up delivery of the placenta. |
| 10 | Turkish respondent said that she had told her GP that she suffered from pains in her breast. The GP told her she should get some rest and relax: for example, walk outside. After a while, she was referred to the hospital and was diagnosed with breast cancer and needed a mastectomy. For the respondent, it was still a very painful memory that her complaints had not been not taken seriously. |
| 11 | Turkish respondent said she had had problems with her thyroid, and saw two different doctors at two different hospitals. One doctor prescribed a higher dose of the medication than did the other doctor. The respondent believed that this difference was not good for the treatment, but only after a long period did she discuss it with the doctors. |
| 12 | Turkish respondent said she had eaten something bad and was brought to the hospital with a stomach ache and a lot of vomiting. Her stomach was then operated upon. Afterwards, the respondent did not understand why she needed an operation for something as simple as food poisoning. |
| 13 | Turkish respondent related that she had gone to the Emergency Room because she felt extremely nauseous and thought she had eaten something poisonous. To her surprise, she had to wait 5 hours before she was seen by a physician; during that time she had been extremely anxious because she felt the poison should be “taken out” as soon as possible. |
| 14 | Turkish respondent said she had had an operation and was given a small injection in her stomach by a nurse with little education or experience. The spot where she was injected turned black. She was very frightened and believed something very bad had happened. The nurses reacted in her eyes indifferently, and said that this could happen. Still no one wanted to give her the name of the nurse who gave the injection, and the respondent felt this was strange. |
| 15 | Turkish respondent said she had had a small operation on her hand and was given local anaesthesia. After one injection, however, she still was in pain. She was given another injection but then began to behave in a strange way, singing and behaving in a bizarre manner. After the operation, she wondered what she had been given in the second injection. The doctors said they had not done anything different with the second injection. |
| 16 | Turkish respondent said she had had severe headaches for years, and that the GP only advised her to take paracetamol. After 18 years of headaches, she was finally sent to a specialist and had a MRI scan, but nothing unusual was found. She then went to a doctor in Turkey. The doctor asked about the contraceptive she was using, and said that the pills she was taking could cause headaches. She stopped taking the pills and her headaches went away. The respondent thought it was strange that the Dutch specialist and GP never considered the connection between her headache and the contraceptive pills, even though she had taken the box of pills with her to show the specialist. |
| 17 | Italian respondent told he was diagnosed with prostate cancer, but refused to believe this diagnosis because doctors ‘have screwed up’ before (the diagnosis of Mexican flue had been overlooked earlier that year and the respondent ended up in the hospital). The respondent felt he could not trust his doctor anymore and refused the suggested radiotherapy. |
|  | Situation 3: Respondents felt excluded from optimal care because care providers were prejudiced or discriminate |
| 18 | Chilean respondent was in the Netherlands illegally, and was uninsured. He suffered from an extensive infection between his nose and his right eyes. He went to the hospital but was turned away because he was not insured, even though in the Netherlands uninsured patients are entitled to medical care. A few months later, another hospital accepted him as a patient. The respondent was diagnosed with cancer, and his eye had to be removed. |
| 19 | Turkish respondent said she had had an operation on her uterus, but was still in a lot of pain. She visited a gynecologist in Turkey who found a bacterial infection. Back in the Netherlands, she had another examination, confirming the finding of the bacteria. She had treatment but was still in pain. She then told the Dutch gynecologist that she had visited a Turkish doctor. The Dutch gynecologist responded that “this was proof she did not trust him anymore”. The respondent felt this was not the case; she had only wanted the gynecologist to admit that he had overlooked the bacteria. |
| 20 | Turkish respondent said she had had injections in her shoulder because of a painful arm that she hardly could use anymore. When she was in Turkey on holiday she also went to see a doctor. He told her that she should not have too many injections because it was bad for her bones. When she was back in the Netherlands she told this to the Dutch specialist, and he sent her back to Turkey. She felt abandoned and mistreated by the Dutch specialist. |
| 21 | Surinamese respondent had had postoperative complaints that were not taken seriously; two days after the operation it was discovered he had a perforated oesophagus and had to be transferred to Intensive Care. In the interview, his daughter explained that she thought care providers’ prejudice concerning how ethnic minorities express pain may have played a role in their neglect of the respondent’s complaints. |
| 22 | Turkish respondent said she had had an operation on her left arm but still was in pain afterwards, and the arm was much bigger than the other one. While she was in Turkey on holiday, she felt that the pain was less, probably because of the warmer climate. When she told this to her doctor, he said she should move back to Turkey. She felt shocked and abandoned as a result of the doctor’s statement. |
